# Comparison of the Completeness of Spontaneously Reported Adverse Drug Reactions by Consumers, Healthcare Professionals, and Pharmaceutical Companies: An Evaluation of Databases From Two High‐Income Countries

**DOI:** 10.1002/prp2.70164

**Published:** 2025-08-08

**Authors:** Mohammed Gebre Dedefo, Gizat M. Kassie, Eyob Alemayehu Gebreyohannes, Renly Lim, Elizabeth Roughead, Lisa Kalisch Ellett

**Affiliations:** ^1^ Quality Use of Medicines and Pharmacy Research Centre, UniSA Clinical and Health Sciences University of South Australia Adelaide Australia; ^2^ South Australian Health and Medical Research Institute Adelaide South Australia Australia; ^3^ College of Medicine and Public Health Flinders University Adelaide South Australia Australia; ^4^ Centre for Optimisation of Medicines, School of Allied Health The University of Western Australia Perth Australia

**Keywords:** adverse drug reaction reporting systems, drug monitoring, drug safety, drug‐related side effects and adverse reactions, pharmacovigilance

## Abstract

This study assessed whether the completeness of spontaneously reported adverse drug reaction (ADR) reports differs between consumers and healthcare professionals when submitted directly to regulators, and how this compares to reports from pharmaceutical companies. ADR reports (2014–2023) were obtained from public databases in Canada and the United Kingdom (UK), focusing on the medicine classes sodium‐glucose cotransporter 2 inhibitors, glucagon‐like peptide 1 receptor agonists, and dipeptidyl peptidase‐4 inhibitors. ADR report completeness was assessed using vigiGrade tool variables. Descriptive statistics and chi‐square tests were used for analysis. A total of 17 897 reports were analyzed—13 613 from the UK Yellow Card Scheme and 4284 from Canada. Most Canadian reports were submitted by pharmaceutical companies (55%), while in the UK, healthcare professionals submitted the majority (69%). Few reports were submitted directly by consumers in either Canada (4%) or the UK (7%). In Canada, the average completeness was 82% for consumer and healthcare professional reports and 57% for pharmaceutical companies. In the UK, completeness was 80% (consumers), 82% (healthcare professionals), and 69% (pharmaceutical companies). Canadian pharmaceutical company reports were significantly less complete for age, sex, outcome, dose, indication, and route of administration (all *p* < 0.001). In the UK, they were less complete for age, sex, and route of administration (all *p* < 0.001). In conclusion, reports submitted directly to regulators by consumers and healthcare professionals were more complete than those from pharmaceutical companies. The low consumer reporting rate, yet high completeness rate, highlights the need to encourage direct reporting to regulators to improve medicine safety monitoring.

## 1 Introduction

Spontaneous adverse drug reaction (ADR) reporting systems are a fundamental component of pharmacovigilance, providing a widely used and cost‐effective methodology for collecting data on suspected ADRs throughout a medicine's lifecycle [1, 2]. ADR reporting by healthcare professionals, consumers, and pharmaceutical manufacturers assists in the detection and management of ADRs and contributes to the safe use of medicines [3, 4, 5]. When spontaneous ADR reports are submitted, causality assessment is undertaken to determine whether the reported ADR is likely caused by the suspected medicine. Causality assessment aims to assess the causal relationship of the ADR to the medicine at both the individual case level and the population level [6, 7]. It involves analyzing factors such as the patient characteristics, reported ADR symptoms and findings, information about the suspected medicine, timing of the event, dechallenge and rechallenge outcomes, and examining alternative explanations [6, 8]. The completeness of the information included in individual ADR reports is an important factor when undertaking causality assessment of reported reactions with suspected medicines [9]. Similar ADRs reported across multiple individuals are evaluated through disproportionality analysis, case series analysis, pharmacoepidemiologic studies, and other methods to determine if a confirmed causal relationship exists that warrants regulatory action [6, 10]. Verified signals may lead to updates in drug labeling or other public health interventions to support safe medicine use across diverse populations [10].

A systematic review conducted in 2021, involving 11 studies that compared patient‐reported ADRs with medical records (seven studies) and healthcare professional reports (four studies), showed that patients frequently identify unique ADRs not captured by other sources [11]. Between 6% and 66% of ADRs reported by patients were not documented in their medical records, and 11% to 35% were not identified through voluntary reporting by healthcare professionals [11]. These results indicate that patients can provide relevant safety and ADR information about medicines that may not be recorded by other surveillance mechanisms.

Prior studies have shown that the incompleteness of individual spontaneous ADR reports can pose a challenge to determining the causality between reported ADRs and a suspected medicine at both the individual case level and the population level [12, 13, 14, 15]. For example, a study conducted using spontaneous ADR reports in Brazil found that among 820 ADR reports submitted by manufacturers and 179 reports submitted by health centers between 2013 and 2014, only 4% included sufficient evidence to perform a causality assessment for the suspected medicine and the reported ADR [12]. In the Netherlands, a study that compared the completeness of information in ADR reports made by patients and healthcare professionals revealed that clinical information was documented well enough to enable causality assessment in 54% of cases reported by patients and in 63% of cases reported by healthcare professionals [13]. A study conducted in Spain, which included 824 ADR reports recorded in 2014, assessed the completeness of reports using the vigiGrade completeness tool [14]. This study found that completeness varied by reporter type, with 55% of reports by healthcare professionals including sufficient information for causality assessment, but only 23% of reports from pharmaceutical companies including sufficient information [14]. These studies indicate that the completeness of spontaneous ADR reports is often insufficient for subsequent causality assessment of the association between exposure to medicines and suspected ADR.

Consumers and healthcare professionals may submit ADR reports either directly to the regulators or indirectly via pharmaceutical companies. However, it is unclear whether prior studies comparing the completeness of ADR reports by consumers and healthcare professionals considered reports submitted directly to regulators, or if they also included reports submitted indirectly via pharmaceutical companies. This distinction is important because previous studies have indicated that ADR reports submitted through pharmaceutical companies often differ in completeness compared to those submitted directly to regulators, with higher completeness observed in reports provided directly to regulators [12, 16]. When a consumer or healthcare professional reports an ADR to a pharmaceutical company, the company is legally required to submit the report to the regulator. However, during this process, information for causality assessment may be lost or omitted, or may not have been provided by the original reporter. Therefore, comparing the completeness of ADR reports submitted by consumers or healthcare professionals without distinguishing between those submitted directly and those submitted indirectly to medicines regulators may be misleading if differences in completeness are due to limitations in the collection and transmission process.

This study aimed to determine whether the completeness of ADR reports differed between consumers and healthcare professionals when submitted directly to regulators; whether completeness differed from reports submitted by pharmaceutical companies. We considered ADR reports for glucose lowering medicines: sodium‐glucose cotransporter 2 inhibitors (SGLT‐2is), glucagon‐like peptide 1 receptor agonists (GLP‐1RA) and dipeptidyl peptidase‐4 inhibitors (DPP‐4i) as a case study example to answer our research question. We chose these medicines because they are widely used worldwide and had similar regulatory approval timelines across different countries [17, 18, 19, 20].

## 2 Methods

### 2.1 Data Sources, Study Design, and Sample Selection

A retrospective longitudinal analysis was conducted using publicly accessible ADR reports from the Canada Vigilance Adverse Reaction Online Database [21] and the Yellow Card Scheme of the United Kingdom (UK) [22]. We used the generic names of medicines from the class of SGLT‐2is, GLP‐1RA, and DPP‐4i (Data S1) to identify ADR reports for these medicines in the databases. We included reports submitted directly by consumers or by healthcare professionals to these regulators and reports submitted by the pharmaceutical companies between January 1, 2014, and December 31, 2023. We limited the analysis to spontaneous reports. ADR reports derived from research studies and published literature were excluded. Additionally, reports with a missing reporter type were excluded.

Duplicate reports were excluded from the analysis. In the Canada Vigilance ADR reporting database, duplicate reports are designated as duplicates by a specific variable: the Link/Duplicate report information field [23]. In reports with this designation in the Canada Vigilance ADR reporting database, the most recent report was selected and included in the analysis, while other duplicates were excluded. Duplicate reports in the UK Yellow Card Scheme are removed by the regulator prior to making the reports publicly available [24]; thus, this step was not required for the UK data.

### 2.2 Study Variables and Completeness Assessment

The vigiGrade completeness score is a validated tool developed by World Health Organization‐Uppsala Monitoring Centre (WHO‐UMC) to assess completeness of ADR reports [25]. We identified the variables used in the vigiGrade completeness score [25] within the study databases and summarized their availability in Table 1, including variables important for causality assessment as outlined by the WHO [9]. The vigiGrade score, which ranges from 0.07 to 1, imposes penalties for missing information, with major deductions for the absence of time‐to‐onset (50%), age, sex, outcome, or indication (30%), and other variables (10%) (Data S1). Our initial assessment of the ADR reports found that the vigiGrade completeness score could not be calculated for this study due to the absence of essential variables in the data sources. Therefore, an alternative approach was used to assess the completeness of ADR reports [16]. The percentage of reports by consumers, healthcare professionals, and pharmaceutical companies in each dataset with each variable reported was calculated, and the average overall completeness score for reports by consumers, healthcare professionals, and pharmaceutical companies was also calculated for each dataset. The total number of variables recorded in a given dataset by consumers, healthcare professionals, and pharmaceutical companies was used as the denominator for the average overall completeness score calculation, and the number of variables with data recorded was the numerator. Results were expressed as a percentage. For example, in the Canada ADR reporting database, we considered six variables in the completeness assessment (Table 1) and if the report included data for five of those six variables, an average overall completeness score of 83% was calculated. The average overall completeness scores, expressed as percentages, were calculated for reports by consumers, healthcare professionals, and pharmaceutical companies within each database. Additionally, the proportion of ADR reports by consumers, healthcare professionals, and pharmaceutical companies containing data for each variable included in the analysis (Table 1) was determined.

**TABLE 1:** List of variables included for assessing completeness of ADR reports.

Variables		Canada	UK
Variables in the vigiGrade completeness tool^a^	Time‐to‐onset	X	X
	Indication	✓	X
	Outcome	✓	✓
	Sex	✓	✓
	Age	✓	✓
	Dose	✓	X
	Comments	X	X
Additional variable assessed for completeness	Route of administration	✓	✓

*Note:* ✓: The data is available in the publicly accessible ADR report database of that country. X: The data is unavailable in the publicly accessible ADR report database for that country.

^a^ Primary reporter, country, and report type are included in the vigiGrade completeness tool but were omitted from the table because complete information on the primary reporter was part of our inclusion criteria; both data sources contained only domestic reports making the country variable non‐informative, and only spontaneous ADR reports were considered for report type.

The primary outcome of this study was a comparison of the completeness of ADR reports submitted directly to the regulator by consumers and healthcare professionals. The secondary outcome was a comparison of the completeness of ADR reports submitted directly to the regulator by consumers or healthcare professionals with the completeness of reports submitted via pharmaceutical companies.

### 2.3 Data Analysis

Descriptive statistics were used to describe the proportion of ADR reports submitted by consumers, healthcare professionals, and pharmaceutical companies and the completeness of reports by each reporter type. Results were stratified by database (Canada, UK). A chi‐square test was conducted to determine whether statistically significant differences existed between the completeness of reports for age, sex, outcome, indication, dose, route of administration, and whether the primary reporter was a consumer or healthcare professional, and whether they were submitted directly to the regulator by consumers or healthcare professionals or indirectly via pharmaceutical companies. The analysis was performed using the Statistical Package for Social Sciences (SPSS) version 30 (IBM Corporation, New York, USA).

## 3 Results

**FIGURE 1:**
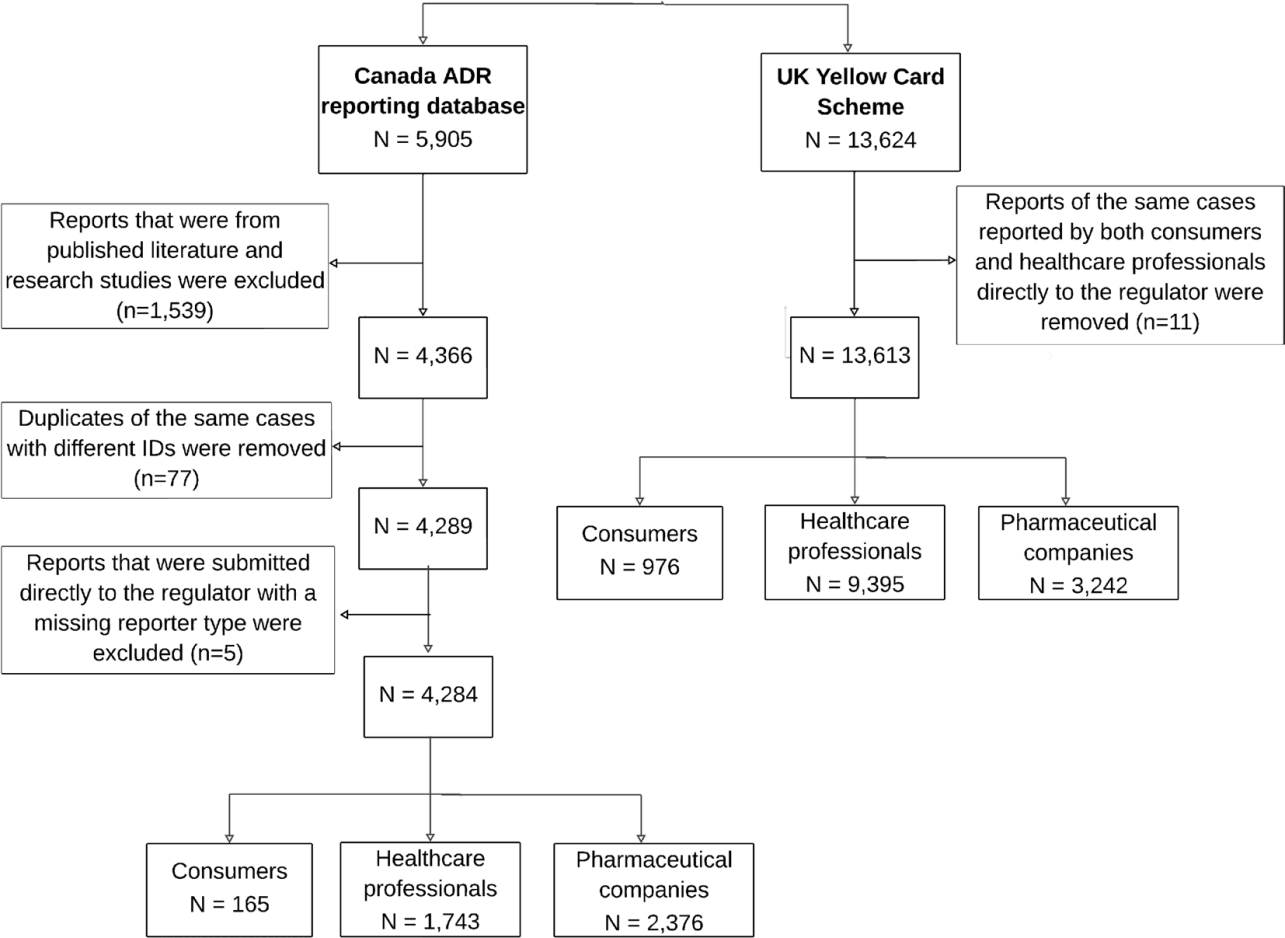
Flowchart of selection of the cases and exclusion criteria of ADR reports involving SGLT‐2i, GLP‐1RA, and DPP‐4i medicines from 2014 to 2023 in Canada and the UK.

A total of 17 897 individual case safety reports submitted by consumers, healthcare professionals, and pharmaceutical companies were analyzed; 13 613 reports were from the UK Yellow Card Scheme and 4284 reports were from the Canadian ADR reporting database (Figure 1). In the UK database, 976 (7%) reports were submitted by consumers and 9395 (69%) by healthcare professionals directly to the regulator, with 3242 (24%) reports submitted via pharmaceutical companies. In the Canadian database, consumers submitted 165 (4%) reports and healthcare professionals submitted 1743 (41%) reports directly to the regulator, with the remaining 2376 (55%) reports submitted via pharmaceutical companies (Figure 1).

### 3.1 Completeness of Information of ADR Reports Submitted Directly to the Regulator by Consumers and Healthcare Professionals

**FIGURE 2:**
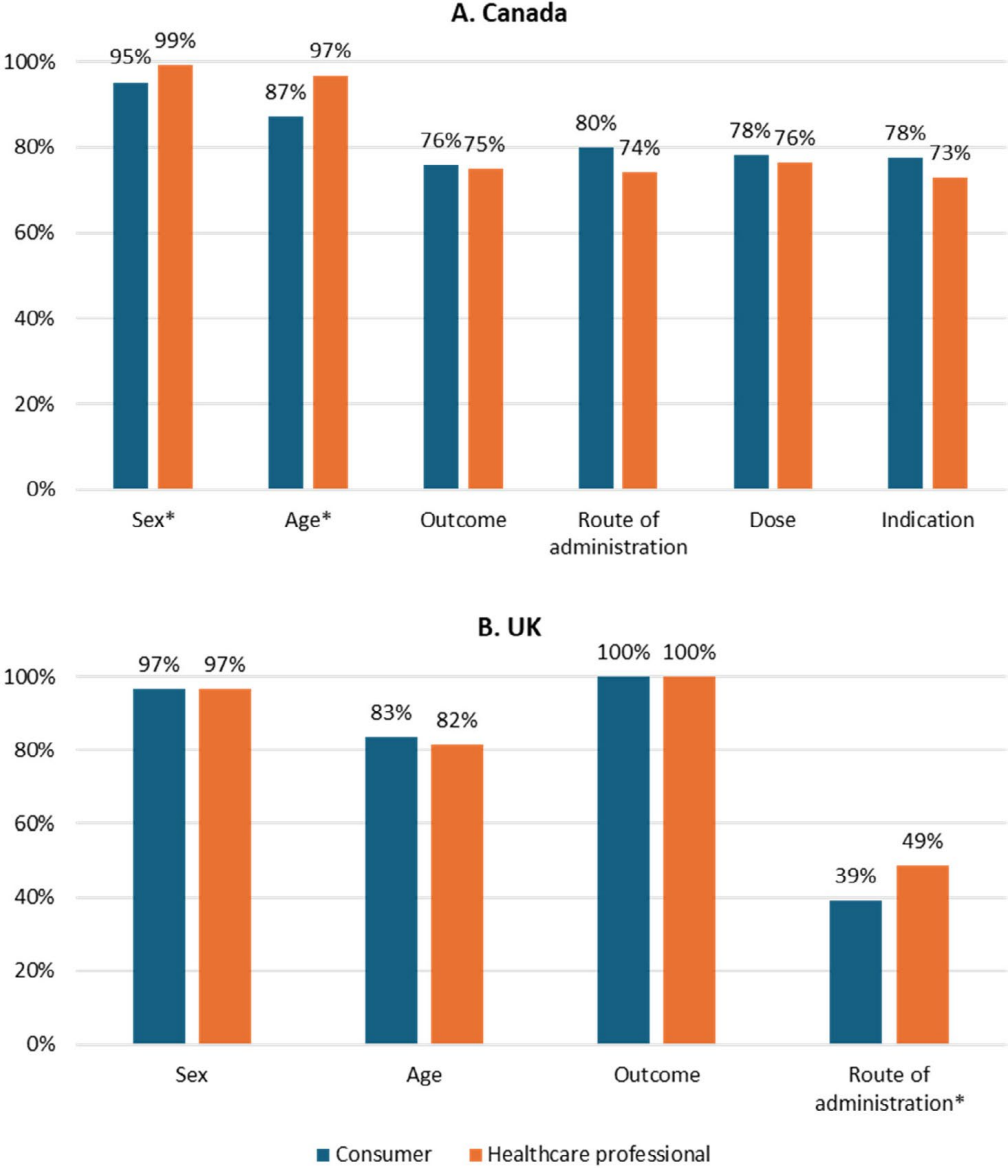
Proportion of ADR reports which included data for specific variables in ADR reports by consumers and healthcare professionals in Canada and the UK. *Statistically significant difference in proportion of reports with complete information for this variable by reporter type. Where values are not displayed, the data is not reported in the publicly available data for that country. ADR, adverse drug reaction; UK, United Kingdom.

In both countries, sex was completed in more than 95% of the reports submitted by both consumers and healthcare professionals, and age was completed in more than 80% of reports by both reporter types. Information on outcomes was complete in all reports submitted by consumers and healthcare professionals in the UK, and information on outcomes was complete in three quarters of reports submitted by consumers and healthcare professionals in Canada. The variable with the lowest completeness in the UK dataset was route of administration, with 39% of reports (*n* = 383) by consumers and 49% of reports (*n* = 4564) by healthcare professionals including this information. The variable with the lowest completeness in the Canadian dataset was indication; however, the completeness of reporting of this variable was still relatively high, with 73% of reports (*n* = 1273) by healthcare professionals and 78% of reports (*n* = 128) including this information (Figure 2).

Comparison of completeness for reporting of different variables by consumers and healthcare professionals using the chi‐square test showed no significant differences for outcome, dose, indication, and route of administration in the Canadian database, and for age, sex, and outcome in the UK database. In Canada, a higher proportion of healthcare professional reports than consumer reports reported age (97% vs. 87%; *p* < 0.001) and sex (99% vs. 95%; *p* < 0.001). Similarly, in the UK, a higher proportion of reports by healthcare professionals compared to consumers reported route of administration (49% vs. 39%; *p* < 0.001).

### 3.2 Comparison of Completeness of Reporting of Specific Variables in ADR Reports Submitted by Consumers, Healthcare Professionals, and Pharmaceutical Companies to the Regulator

In both the Canadian ADR reporting database and the UK's Yellow Card Scheme, the completeness of reporting specific variables was higher when ADR reports were submitted directly to the regulator by consumers or healthcare professionals compared to reports submitted via pharmaceutical companies. In the Canadian dataset, less than half of all reports submitted via pharmaceutical companies included information relating to route of administration, outcome, dose, or indication (Table 2).

**TABLE 2:** Comparison of the proportion of ADR reports submitted by consumers, healthcare professionals, and pharmaceutical companies to the regulator with complete information for specific variables.

Variables	Canada			UK		
	Consumer, *n* = 165	Healthcare professional, *n* = 1743	Pharmaceutical company, *n* = 2376	Consumer, *n* = 976	Healthcare professional, *n* = 9395	Pharmaceutical company, *n* = 3242
Age	144 (87.3%)	1687 (96.8%)	1461 (61.5%)*	814 (83.4%)	7661 (81.5%)	1631 (50.3%)*
Sex	157 (95.2%)	1728 (99.1%)	2125 (89.4%)*	944 (96.7%)	9067 (96.5%)	3019 (93.1%)*
Outcome	125 (75.8%)	1306 (74.9%)	1109 (46.7%)*	976 (100%)^a^	9395 (100%)^a^	3242 (100%)^a^
Dose	129 (78.2%)	1331 (76.4%)	1154 (48.6%)*	−	−	−
Indication	128 (77.6%)	1273 (73.0%)	1127 (47.4%)*	−	−	−
Route of administration	132 (80.0%)	1291 (74.1%)	1074 (45.2%)*	383 (39.2%)	4564 (48.6%)	1058 (32.6%)*

*Note:* −, Variable not reported in that database.

^a^ No statistics are computed because the outcome is constant, with 100% completeness for both consumers and healthcare professionals in the UK database.

* *p* < 0.001 for the chi‐squared test (Consumer vs. Healthcare professional vs. Pharmaceutical company).

In the Canadian database, a lower proportion of ADR reports submitted by pharmaceutical companies than those submitted directly to the regulator by consumers and healthcare professionals had information recorded for age (62%, 87%, and 97% of reports respectively; *p* < 0.001), sex (89%, 95%, and 99% respectively; *p* < 0.001), outcome (47%, 76%, and 75% respectively; *p* < 0.001), dose (49%, 78%, and 76% respectively; *p* < 0.001), indication (47%, 78%, and 73% respectively; *p* < 0.001), and route of administration (33%, 39%, and 49% respectively; *p* < 0.001). Similarly, in the UK database, ADR reports submitted by pharmaceutical companies were less likely than those submitted directly to the regulator by consumers and healthcare professionals to include information about age (50%, 83%, and 82% of reports respectively; *p* < 0.001), sex (93%, 97%, and 96% respectively; *p* < 0.001), and route of administration (33%, 39%, and 49% respectively; *p* < 0.001) (Table 2).

### 3.3 Average Overall Completeness Score of ADR Reports by Consumers, Healthcare Professionals, and Pharmaceutical Companies Between 2014 and 2023

The average overall completeness of reports was determined by identifying the number of reports with information reported for all available variables. The average overall completeness score of ADR reports was 82% for reports made by both consumers and healthcare professionals in the Canadian database. The average overall completeness was 80% for reports by consumers and 82% for reports made by healthcare professionals in the UK database. In the Canadian database, all six of the available variables were complete in 53% of the reports by consumers and in 49% of the reports made by healthcare professionals. In the UK database, all four of the available variables were complete in 38% of the reports by consumers and in 47% of the reports by healthcare professionals.

The average overall completeness of reports submitted by pharmaceutical companies was 57% in Canada and 69% in the UK. All six available variables were complete in only 15% of reports submitted via pharmaceutical companies in Canada, and all four variables were complete in 21% of reports submitted via pharmaceutical companies in the UK.

## 4 Discussion

Our study found that spontaneous ADR reports submitted by consumers and healthcare professionals directly to medicines regulators have a high level of completeness of information. The average overall completeness score for ADR reports made by consumers and healthcare professionals was over 80% in both databases. Canadian healthcare professionals were more likely to report information relating to sex and age than consumers, although reporting of these variables was high (> 87% completeness) by both groups. UK healthcare professionals were more likely to report the route of administration than consumers. In both databases, reports submitted directly to the regulator by consumers or healthcare professionals had higher completeness than reports submitted via pharmaceutical companies.

Similar to our findings, previous studies in the United States (US) using the Food and Drug Administration Adverse Event Reporting System (FAERS) database [26], in Germany using the EudraVigilance database [27], and in the Netherlands using the Netherlands pharmacovigilance centre [28] have shown that consumers are more likely to omit information on age and route of administration than healthcare professionals. Although our findings align with the results of studies from the US [26] and Germany [27] regarding the route of administration and age, there is a difference in our estimates of the completeness of indication reporting. While previous studies found that consumers were more likely to report indications than healthcare professionals [26, 27], our study found no difference in completeness between the two groups. This inconsistency may be attributed to the nature of the data used for analysis. In our study, we analyzed ADR reports specifically for selected antidiabetic medicines. In contrast, the study conducted in the US [26] evaluated reports for all medicines within a single year (2016), while the study from Germany [27] focused on ADR reports related to opioid medicines over a substantially longer period (1980–2018).

In the UK, outcome information was complete in all reports, while the route of administration was reported in only 39% of consumer and 49% of healthcare professional reports. In Canada, both outcome and route of administration information were complete in approximately three‐quarters of reports submitted by consumers and healthcare professionals. The difference in the completeness of ADR reports between Canada and the UK could be related to variations in the default ADR reporting formats used in each country. Both countries provide paper‐based (Canada [29] and the UK [30, 31]) and online (Canada [32] and the UK [33]) ADR reporting forms, but the structure and content of these forms differ within each country (i.e., between paper and online formats) as well as between the two countries. These differences in the reporting formats may contribute to the observed variation in the completeness of reports. However, as the method of reporting (paper or online) was not recorded in the dataset used for this study, we were unable to compare the completeness of reports by reporting method.

Despite the observed differences in completeness of reporting of age and sex within the Canadian database and the route of administration in the UK database, our findings indicate no differences in the completeness of reports for outcome, dose, and indication between consumers and healthcare professionals. Previous studies conducted in South Africa using Vigibase [34] and in the Netherlands using the database of the Netherlands pharmacovigilance centre [13] have similarly reported no significant difference in the completeness of ADR reports between consumers and healthcare professionals. This suggests that, although variations exist based on the type of dataset analyzed regarding the completeness of reports for certain variables, consumers provide information that is comparable in terms of completeness to that reported by healthcare professionals when reporting directly to regulators.

The comparable overall completeness of reports submitted by consumers and healthcare professionals in our study indicates that consumers can effectively contribute to the monitoring of medicine safety. Previous studies indicate that consumer reports provide valuable safety insights, often complementing reports from healthcare professionals [35, 36, 37]. However, our findings indicate that the proportion of consumers reporting ADRs is low, accounting for only 4% and 7% of reports in the Canadian and UK databases, respectively. Limited awareness about the reporting process may hinder consumer participation [38, 39, 40]. To address this issue, it is important to promote consumer ADR reporting through awareness initiatives and the implementation of accessible ADR reporting tools [41, 42, 43]. Such measures could enhance both the quantity and quality of reports, ultimately strengthening medicine safety monitoring [44].

Our findings indicate that both consumers and healthcare professionals tend to provide more complete information on demographic details such as sex and age, whereas the route of administration is the least reported variable. It may be that terms like “route of administration” are less well understood by consumers. A previous review study assessing errors related to the route of administration identified that consumers often have difficulty understanding the information on “route of administration,” resulting in errors in 2%–39% of cases [45]. The frequent omission of information on the route of administration in both datasets could also be influenced by how consumers and healthcare professionals report ADRs. For example, they may have referred to the medicine in the ADR report by its name and formulation type, such as “X tablets”, which implies the route of administration of medicine X but does not explicitly state it. As a result, this information might not be recorded in the databases used for this study if it was not reported in a specific ‘route of administration’ variable. The extent to which this may have affected the results of our study could not be determined.

Significant differences were observed in the completeness of ADR reports between those directly submitted to the regulator by consumers and healthcare professionals and those submitted through pharmaceutical companies, with more complete information provided in reports made directly to the regulator. In line with our finding, two studies conducted in Brazil and Spain reported that the completeness of information in ADR reports was higher when directly reported to the regulator compared to indirect reporting through pharmaceutical companies [12, 16]. This may be related to the use of standardized ADR reporting forms by consumers and HCPs when submitting reports to regulatory bodies. In contrast, the information collected by pharmaceutical companies may not follow such a standardized format, potentially resulting in incomplete information for subsequent reporting to regulatory bodies [46]. Additionally, pharmaceutical companies have a mandatory requirement to submit reports to regulators based on the information they receive, even if it is incomplete, which may result in less complete reports. Our findings suggest that encouraging direct reporting to the regulator by consumers and healthcare professionals would improve the completeness of ADR reports, supporting better causality assessments between medicines and reported ADRs.

While this study incorporated variables recorded in the publicly accessible databases of both Canada and the UK, based on the variables included in the vigiGrade completeness tool, this study has certain limitations. Specifically, the use of a publicly accessible database means that variables such as the medicine start date and event start date, which are required for determining time‐to‐onset, a variable essential for causality assessment, were not included and thus not analyzed. Additionally, the databases did not specify the reporting methods used (e.g., paper forms, phone calls, or digital tools), preventing an assessment of how reporting methods impact ADR report completeness.

## 5 Conclusion

The completeness of ADR reports submitted by consumers and healthcare professionals directly to regulators showed that healthcare professionals provided more complete information on age, sex, and route of administration. However, the overall completeness of reports from both consumers and healthcare professionals was comparable and high, suggesting that consumers can complement healthcare professionals in providing complete ADR reports, which are essential for causality assessments between medicines and reported ADRs. Reports made by consumers or healthcare professionals directly to regulators were more complete than those submitted via pharmaceutical companies, highlighting the importance of direct reporting. The proportion of reports submitted by consumers directly to regulators was low, highlighting the necessity to encourage consumers to report ADRs directly to regulators to enhance medicine safety monitoring.

## Author Contributions

M.G.D. contributed to the conception and design of the study, data analysis, data interpretation, drafting the manuscript, and critically revising the manuscript; L.K.E. and G.M.K. contributed to the conception and design of the study, data interpretation, and critically revising the manuscript; E.A.G. contributed to the design of the study, data interpretation, and critically revising the manuscript; R.L. contributed to the conception and design of the study and data interpretation; and E.R. contributed to data interpretation and critically revising the manuscript. All authors read and approved the final manuscript.

## Ethics Statement

The authors have nothing to report.

## Consent

The authors have nothing to report.

## Conflicts of Interest

The authors declare no conflicts of interest.

## Supporting information


**Data S1:** prp270164‐sup‐0001‐DataS1.docx.

## Data Availability

The data that support the findings of this study are available from the corresponding author upon reasonable request.
