# Supplementary material for: Comparison of the Completeness of Spontaneously Reported Adverse Drug Reactions by Consumers, Healthcare Professionals, and Pharmaceutical Companies: An Evaluation of Databases From Two High‐Income Countries
Source: Pharmacol Res Perspect. 2025 Aug 8;13(4):e70164. doi: 10.1002/prp2.70164 (PMC12332888; doi:10.1002/prp2.70164)
Supplement: Supplementary file 1 — Data S1: prp270164‐sup‐0001‐DataS1.docx. [file PRP2-13-e70164-s001.docx]

**Supplementary material 1**

**Table S1**: Medicines included in the ADR report analysis.

| **SGLT-2is** | **GLP-1RA** | **DPP-4i** | **Medicines in fixed-dose combination form** |
| --- | --- | --- | --- |
| Canagliflozin | Dulaglutide | Sitagliptin | Alogliptin/Metformin |
| Dapagliflozin | Liraglutide | Saxagliptin | Alogliptin/Pioglitazone |
| Empagliflozin | Semaglutide | Linagliptin | Canagliflozin/Metformin |
| Ertugliflozin | Exenatide | Alogliptin | Dapagliflozin/Metformin/Saxagliptin |
| Sotagliflozin | Lixisenatide | Vildagliptin | Dapagliflozin/Saxagliptin |
|  |  |  | Empagliflozin/Linagliptin |
|  |  |  | Empagliflozin/Linagliptin/Metformin |
|  |  |  | Ertugliflozin/Sitagliptin |
|  |  |  | Linagliptin/Metformin |
|  |  |  | Linagliptin/Pioglitazone |
|  |  |  | Metformin/Saxagliptin |
|  |  |  | Metformin/Sitagliptin |
|  |  |  | Metformin/Vildagliptin |
|  |  |  | Empagliflozin/Metformin |
|  |  |  | Dapagliflozin/Metformin |
|  |  |  | Ertugliflozin/Metformin |

**Supplementary material 2**

**Table S2**: Dimensions accounted for in the vigiGrade Completeness score with penalties applied.

| **Dimension** | **Description** | **Considerations** | **Penalty** |
| --- | --- | --- | --- |
| **Time-to-onset** | Time from treatment start to the suspected ADR. | Imprecise information is penalized if there is ambiguity as to whether the drug preceded the adverse event; with 30% if the uncertainty exceeds 1 month, 10% otherwise. | 50%  30%  10% |
| **Indication** | Indication for treatment with the drug | Penalty imposed if the information is missing or cannot be mapped to standard terminologies such as ICD or MedDRA. | 30% |
| **Outcome** | Outcome of suspected ADR in the patient. | "Unknown" treated as missing. | 30% |
| **Sex** | Patient sex. | "Unknown" treated as missing. | 30% |
| **Age** | Patient's age at onset of the suspected ADR. | Age "unknown" is treated as missing. 10% penalty is imposed if only the age group is specified. | 30%  10% |
| **Dose** | Dose of the drug(s). | Penalty imposed if the total daily dose cannot be calculated from the included fields. | 10% |
| **Country** | Country of origin. | Supportive in causality assessment since medical practice and adverse reaction reporting vary between countries. | 10% |
| **Primary reporter** | Occupation of the person who reported the case (e.g. Physician, Pharmacist). | Supportive in causality assessment since the interpretation of reported information may differ depending on the reporter's qualifications "Unknown" penalized as missing information, whereas "Other" is not penalized. | 10% |
| **Report type** | Type of report (e.g. spontaneous report, report from study, other). | "not available to sender (unknown)" treated as missing. | 10% |
| **Comments** | Free text information. | Uninformative text snippets excluded. | 10% |
